# Uncovering the Hidden Threat: Ileocolic Intussusception in an Adult With Appendicular Tumor

**DOI:** 10.7759/cureus.72809

**Published:** 2024-10-31

**Authors:** Mrunal Panchal, Shishir Kumar, Khushboo Jha, Kaushik Saha, Abhijit Kundu

**Affiliations:** 1 Department of Surgery, Tata Main Hospital, Jamshedpur, IND; 2 Department of Pathology, Manipal Tata Medical College, Jamshedpur, IND; 3 Department of Pathology, Tata Main Hospital, Jamshedpur, IND

**Keywords:** appendicular mucinous neoplasm, intussusception, lead point, pseudomyxoma peritonei, right hemicolectomy

## Abstract

Intussusception in adults is a rare condition, often associated with an underlying pathological lead point. This case report describes a case of intussusception in a 37-year-old female patient with an unusual lead point: an appendicular mucinous neoplasm. This case highlights the clinical presentation, diagnostic process, and management of adult intussusception caused by an appendicular neoplasm. The patient underwent a right hemicolectomy, with ileo-transverse anastomosis. The lead point was a low-grade appendiceal mucinous neoplasm (LAMN) without any intraoperative spillage. No signs of recurrence were seen till the last follow-up visit. Surgical intervention with appropriate resection of the lead point remains the treatment of choice, with histopathological analysis crucial for further management, in any adult intussusception.

## Introduction

Intussusception in adults represents 5% of all cases of intussusception [1]. It accounts for 1%-5% of bowel obstructions in adults and is typically associated with a pathological lead point, often a tumor that may be benign or malignant [2]. Appendicular mucinous neoplasms are rare and can serve as a lead point [3]. Here, we discuss the clinical presentation and treatment of a 37-year-old female patient with an intussusception secondary to an appendicular mucinous neoplasm. The patient underwent a right hemicolectomy, with ileo-transverse anastomosis. The lead point was a low-grade appendiceal mucinous neoplasm (LAMN). As there was no intraoperative spillage during the resection, no further intervention was needed in this case. The patient has been symptom-free till the last follow-up visit.

## Case presentation

**Figure 1:**
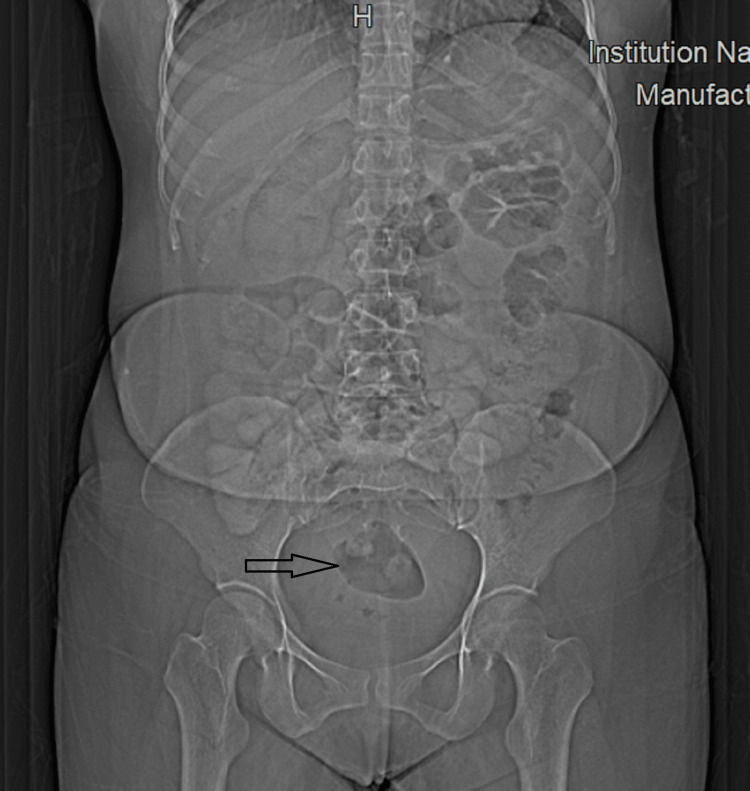
Normal abdominal X-ray pattern of the patient with gas shadow reaching till the rectum marked by asterisk

A 37-year-old lady came to the surgery clinic with complaints of intermittent abdominal pain for the past two weeks, localized mainly in the right lower quadrant. The pain was associated with nausea and intermittent nonbilious vomiting. There was no history of fever, trauma, hematuria, or bleeding per rectum, and menstrual history was normal. There was tenderness in the right iliac fossa on deep palpation, though no lump was felt, and the rest of the systemic examination and vital signs were normal. The X-ray of the abdomen did not show any air fluid levels (Figure 1).

**Figure 2:**
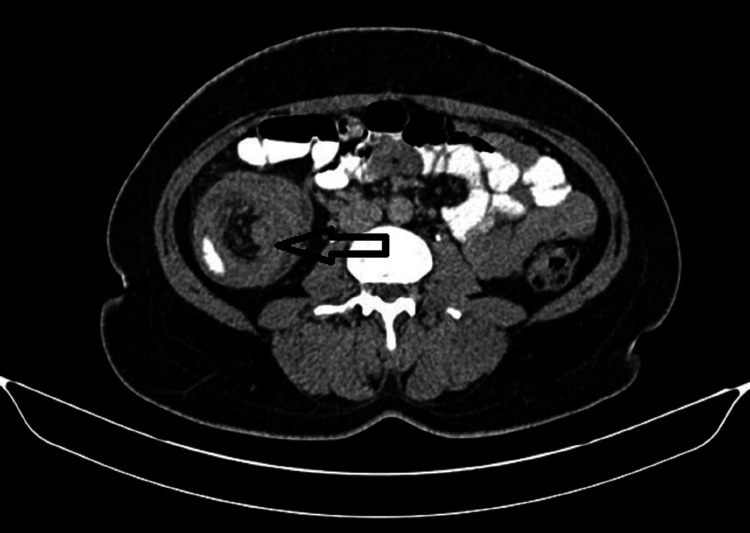
Asterisk showing ileocolic intussusception

**Figure 3:**
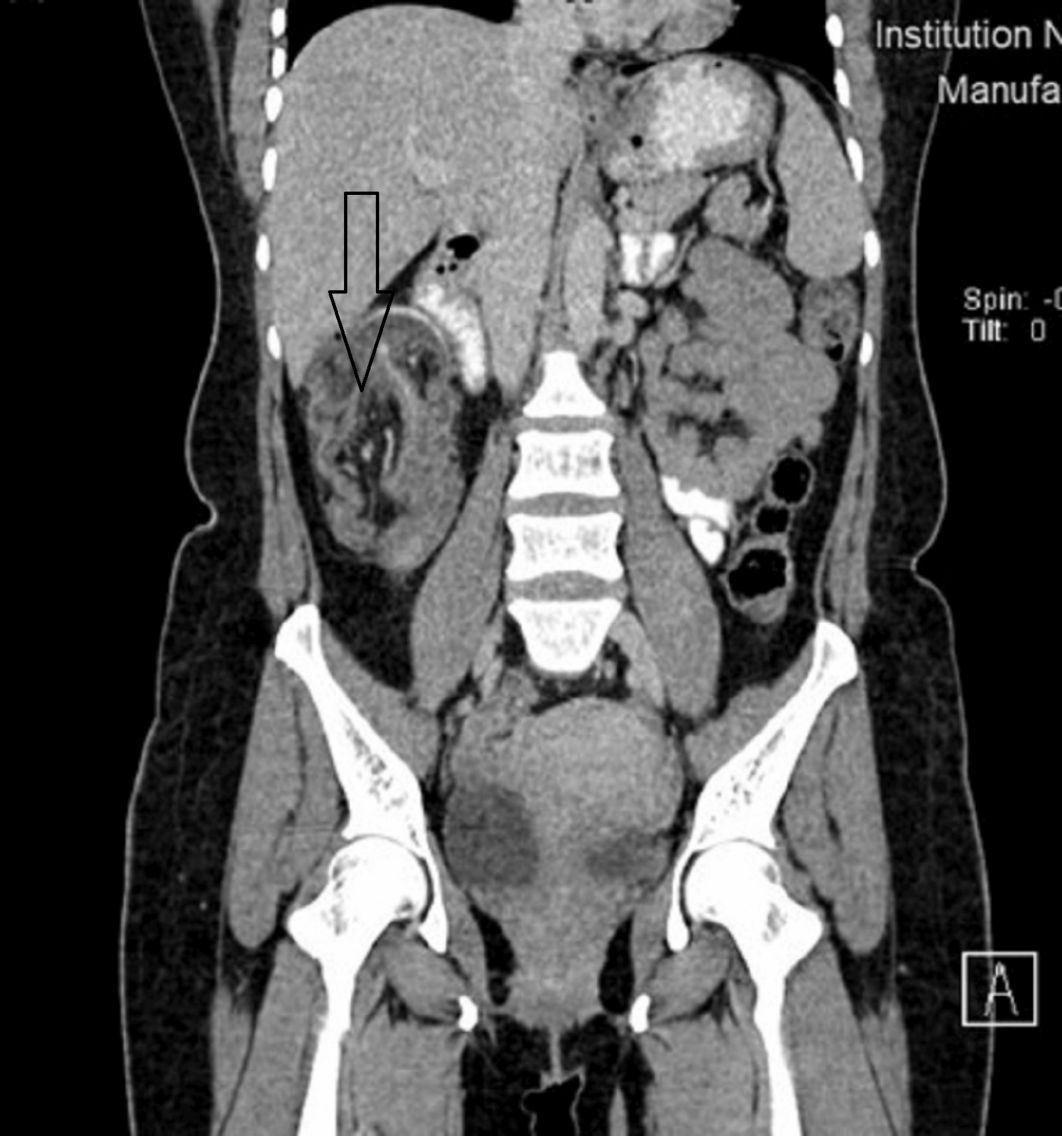
Asterisk showing ileocolic intussusception in the coronal plane

Ultrasonography of the abdomen showed ileocolic intussusception, but no free fluid in the abdomen. Laboratory investigations showed a normal blood count, liver function tests, and renal profile. A contrast-enhanced computed tomography (CT) of the whole abdomen confirmed the ileocolic intussusception (Figures 2-3).

**Figure 4:**
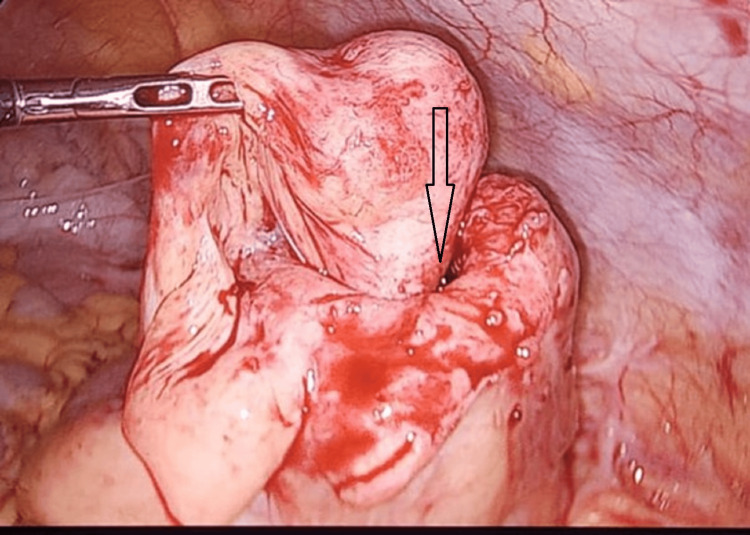
Asterisk showing ileocolic intussusception with the tip of the appendix acting as the lead point

A diagnostic laparoscopy through an umbilical 10 mm camera port was done. An ileocolic intussusception was seen, with intussusceptum noted till the proximal part of the ascending colon (Figure 4).

**Figure 5:**
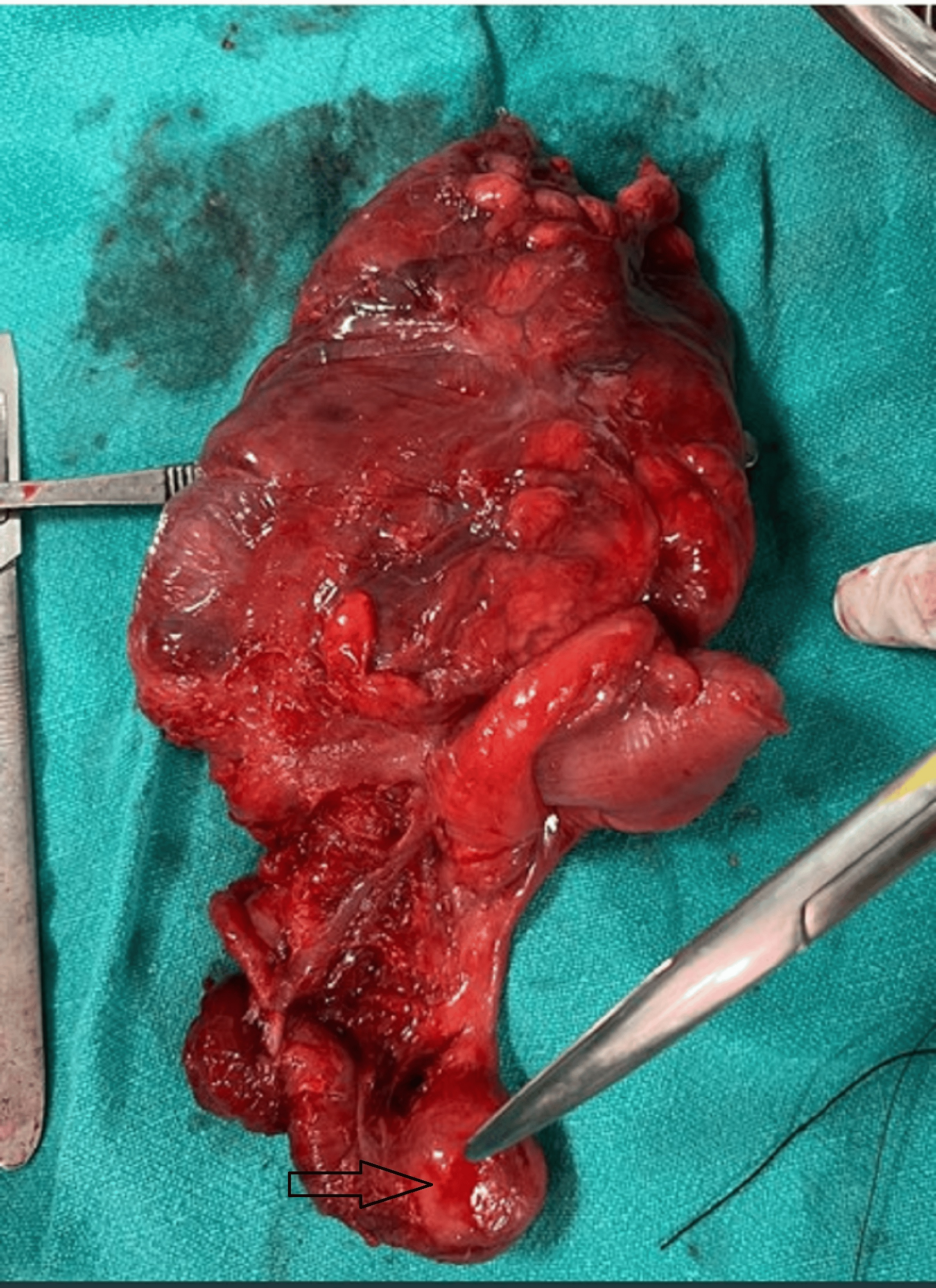
Asterisk showing tumor at the tip of the appendix in the resected specimen

Two more working ports were made in the suprapubic and left iliac fossa regions, and an attempt to reduce the intussusceptum was made. The intussusceptum could not be pulled out, and to avoid the intestinal wall damage, a midline laparotomy incision was made. The lump was firm to hard, and due to inability to reduce the intussusceptum, a right hemicolectomy and ileocolic anastomosis were done (Figure 5). 

**Figure 6:**
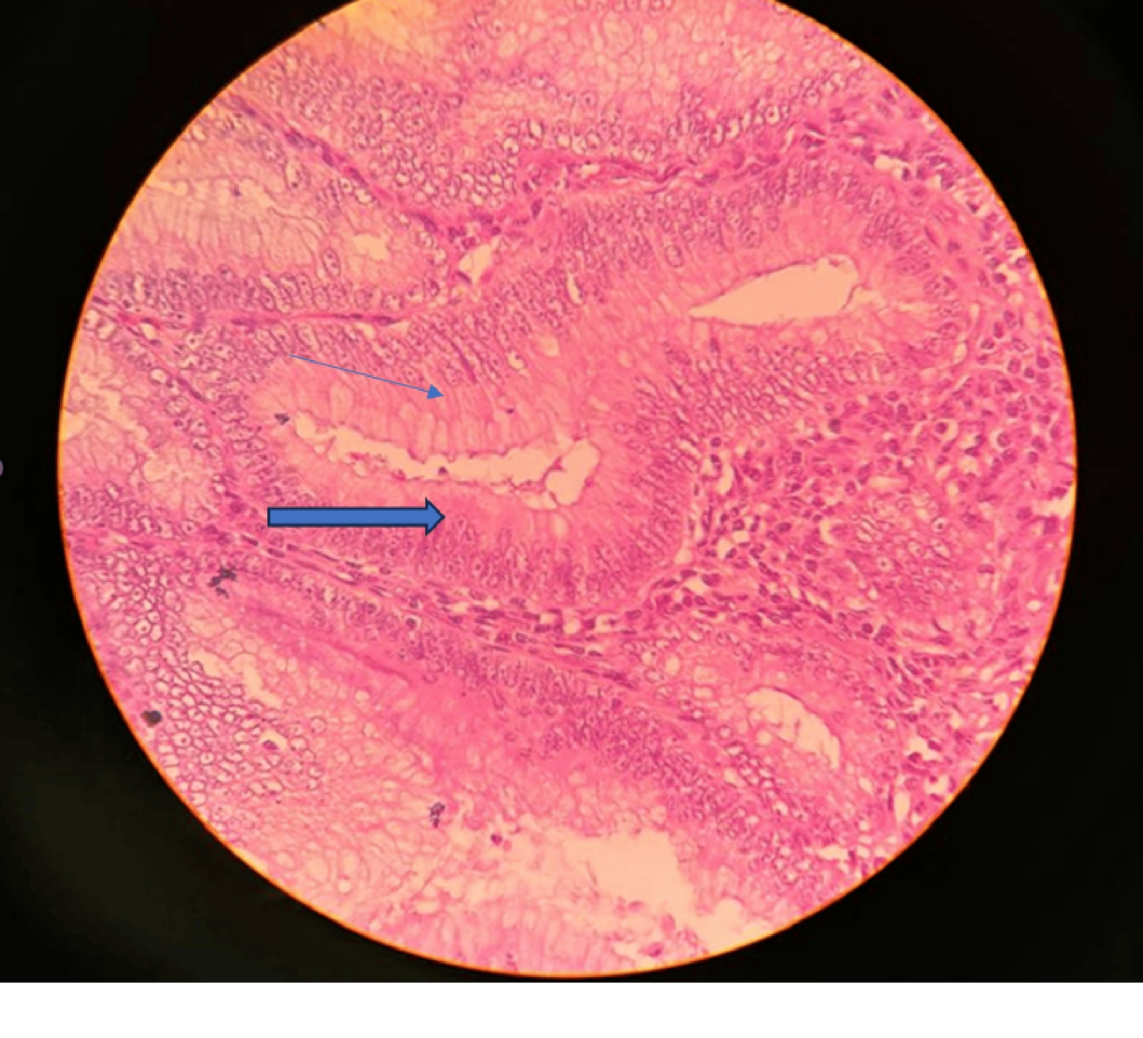
40X view, hematoxylin and eosin section showing mucinous epithelial cells with apical mucin (single arrow) and elongated nuclei with low-grade nuclear atypia (bold arrow)

**Figure 7:**
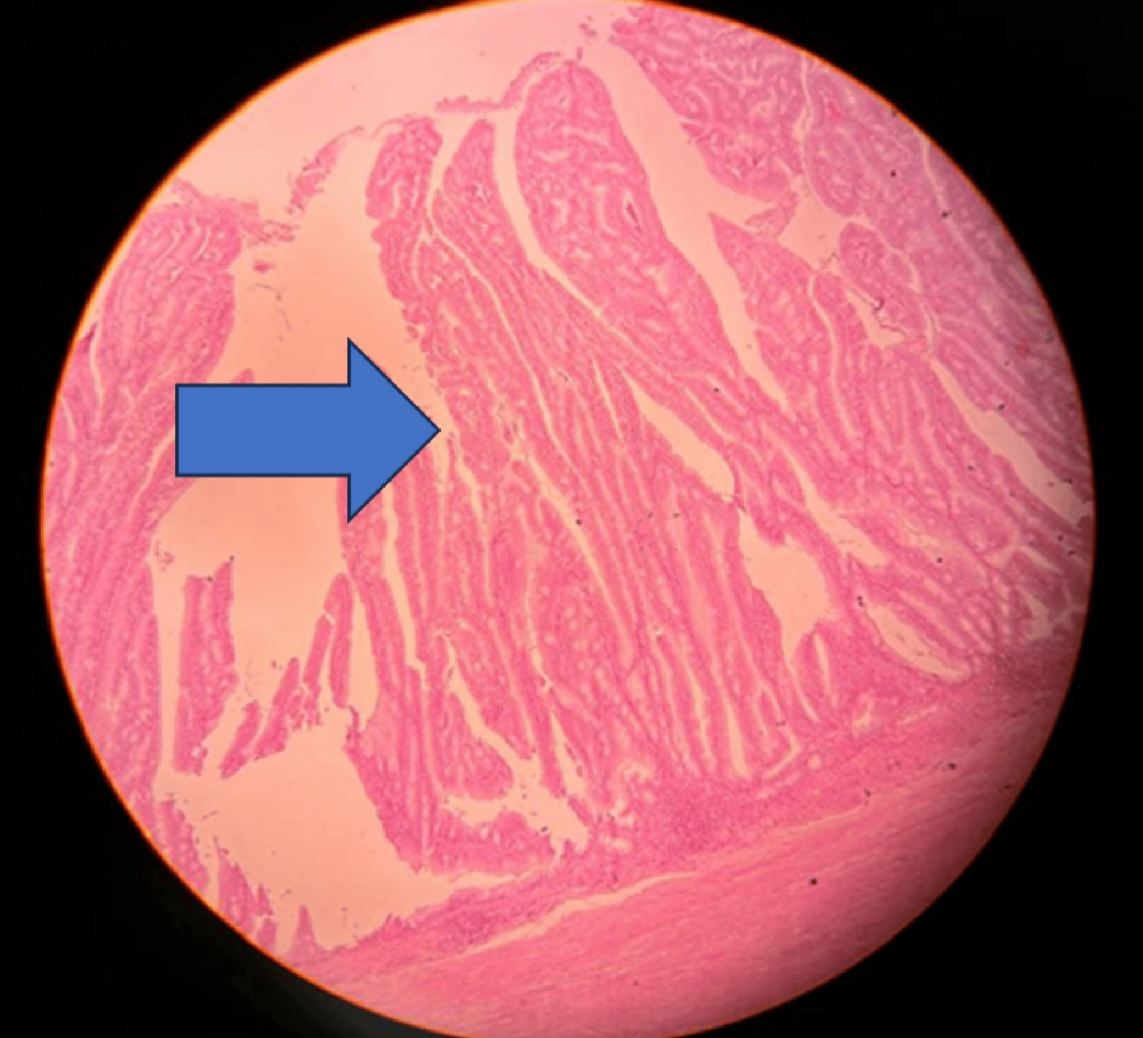
4X view, hematoxylin and eosin section with asterisk showing villous proliferation of mucinous epithelial cells

Enteral feeds were started on the third postoperative day, and the patient tolerated full feeds by postoperative day 5. Histopathology of the resected specimen revealed a LAMN, with villous projection of mucinous epithelial cells from the appendicular lumen with abundant apical mucin, elongated nuclei, and low-grade nuclear atypia (Figures 6-7).

**Figure 8:**
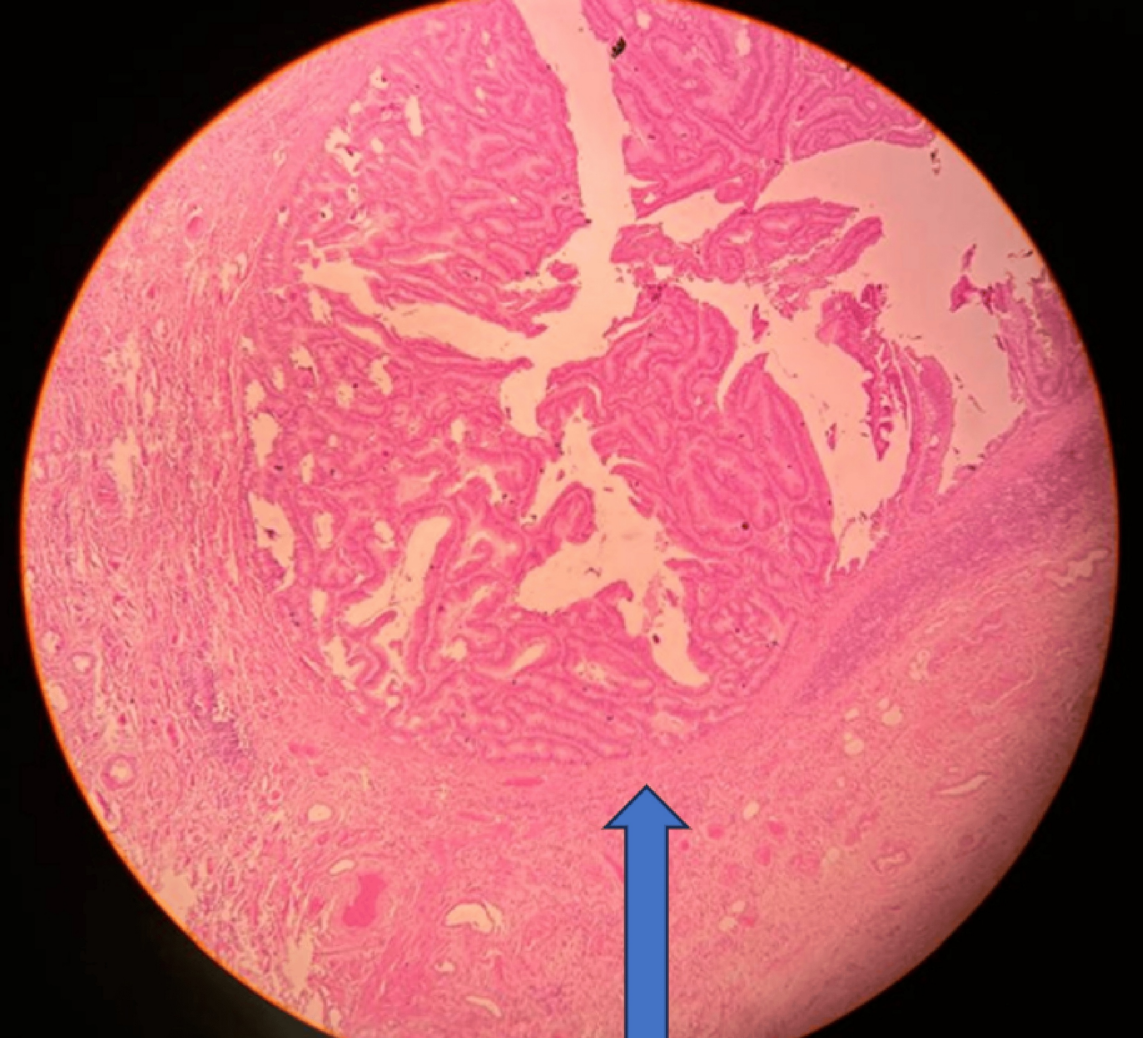
4X view, hematoxylin and eosin section with asterisk showing tumor cells confined to the muscularis mucosa with a pushing-type invasion

The tumor cells were confined to the muscularis mucosa with a pushing-type invasion (Figure 8).

The mesoappendix was free of tumor, and no tumor deposits were found in the regional lymph nodes excised along with the hemicolectomy specimen. The patient has been in follow-up for the last six months, with no signs of recurrence.

## Discussion

Intussusception in adults usually has an identifiable lead point. Neoplasms are a common cause, and appendicular mucinous neoplasms, though rare, can act as a lead point, as in this case. Intussusception in the adult age group requires a high index of suspicion and is best diagnosed with imaging, particularly CT, which can identify intussusception and any associated lead point [4].

The standard treatment for intussusception is surgical resection due to the likelihood of an underlying lead point [5]. In this case, after the failure of manual reduction, a right hemicolectomy was decided due to the adult age group and to ensure complete removal of the lead point. Histopathology confirmed a low-grade mucinous neoplasm, thereby validating the decision of a right hemicolectomy, and underscoring the importance of complete resection to prevent potential complications due to rupture, such as pseudomyxoma peritonei (PMP) [6].

Mucinous neoplasms of the appendix have been classified into mucinous adenoma, LAMN, and appendiceal adenocarcinoma (WHO classification, 2019) [7]. The Peritoneal Surface Oncology Group International (PSOGI) has classified PMP as acellular mucin, low-grade mucinous carcinoma peritonei, peritoneal mucinous carcinomatosis (PMCA), and high-grade mucinous carcinoma peritonei with signet ring cells [8]. In our case, the mucin was confined to the muscularis mucosa with a pushing-type invasion, so it was classified as pTis as per the American Joint Committee on Cancer (AJCC) scheme, with minimal chances of recurrence [9]. Usually, LAMN is an incidental finding, with an incidence of 1% of all appendectomy specimens [10]. Intussusception in adults is a complication of LAMN, as in our case, and is an extremely rare presentation.

The patient has been in follow-up for the last six months, with no signs of recurrence. For LAMN, there are no established protocols for follow-up imaging, as the surgery is usually curative if there is no peritoneal spillage, as was in our case [11]. Patients with mucin spillage may need hyperthermic intraperitoneal chemotherapy or cytoreductive surgery and yearly imaging in the form of CT or MRI to detect recurrence [12].

## Conclusions

This case demonstrates the importance of considering a pathological lead point in adult patients with intussusception. The possibility of a tumor being a lead point mandates that the resection be adequate and without any spillage of the tumor so that the malignancy doesn’t get upstaged. Appendicular mucinous neoplasms, though rare, should be included in the differential diagnosis. Spillage in such cases can lead to PMP, thus necessitating cytoreductive surgery or hyperthermic intraperitoneal chemotherapy. Surgical intervention with appropriate resection remains the treatment of choice. When CT suggests possible malignancy, laparoscopic reduction is not desirable, and primary careful laparoscopic or open resection is advisable. Adequate resection should be the goal, with further interventions, and follow-up visits may be scheduled as per the histopathology report.
